# Simultaneous Diagnosis of Acute Crohn's Disease and Endometriosis in a Patient Affects HIV

**DOI:** 10.1155/2018/1509167

**Published:** 2018-05-09

**Authors:** S. Casiraghi, P. Baggi, P. Lanza, A. Bozzola, A. Vinco, V. Villanacci, F. Castelli, M. Ronconi

**Affiliations:** ^1^Division of General Surgery, Gardone Val Trompia Hospital, Gardone Val Trompia, Italy; ^2^Department of Infectious and Tropical Diseases, University of Brescia, Brescia, Italy; ^3^Institute of Pathology, Brescia Spedali Civili General Hospital, Brescia, Italy

## Abstract

This is the case report of a 45-year-old woman affected by HIV, who was hospitalized for diffuse abdominal pain, constipation, and weight loss present for over one month. A colonoscopy showed the presence of a nontransitable stenosis of the ascending colon. A right hemicolectomy was performed. The histological examination reports CD with outbreaks of endometriosis. CD and the HIV infection may coexist in the same individual and it seems that HIV reduces the relapse rate in IBD patients. CD and intestinal endometriosis can also occur simultaneously. The diagnosis is often only made after surgical resection of the diseased segment. These patients were more likely to have stricturing CD but endometriosis does not seem to impact the natural history of CD.

## 1. Introduction

Crohn's Disease (CD) is a chronic relapsing Inflammatory Bowel Disease (IBD). It is characterized by a transmural granulomatous inflammation which can affect any part of the gastrointestinal tract, most commonly the ileum, colon, or both [1]. The incidence is 6–8% for 100000 and the prevalence is 130–200 per 100000 [2]. It affects both sexes equally, most commonly in the second to fourth decade of life [3].

The etiology may include alterations in the immune system, abnormal cytokine levels, and changes in gut permeability and motility [4]. Clinical signs and symptoms of CD are often subtle and depend on the location and severity of the gastrointestinal involvement as well as inflammatory activity [5].

Endometriosis is defined as the presence of functional endometrial tissue in extrauterine sites and is common in women of reproductive age with an incidence of 5–15% of menstruating women [6]. The reported prevalence of endometriosis is 1–20% in asymptomatic women, 10–25% in infertile patients, and 60–70% in woman with chronic pelvic pain [7].

Endometriosis is distinguished in three different phenotypes: ovarian endometriosis, superficial peritoneal endometriosis, and deep infiltrating endometriosis (DIE). Deep endometriosis was defined arbitrarily as endometriosis infiltrating the peritoneum by >5 mm [7]. DIE includes rectovaginal lesions as well as infiltrative forms that involve structures such as the bowel, ureters, and the bladder. It is the most severe form of the disease with an estimated prevalence of 1% in women of reproductive age and 30–40% of all patients with endometriosis [8].

The clinical presentation can be often nonspecific, by simulating IBD, malignancies, or diverticulitis and can vary from microscopic foci to large space-occupying lesions [8]. Extrapelvic endometriosis affects the GI tract of 5% of women with this condition. The rectosigmoid is the most common site for intestinal endometriosis (70% of all cases) while small bowel involvement, usually confined to the distal ileum, is less frequent (1%–7%) and exclusive localization on the ileum is very rare (1–7%) [9].

## 2. Case Report

**Figure 1:**
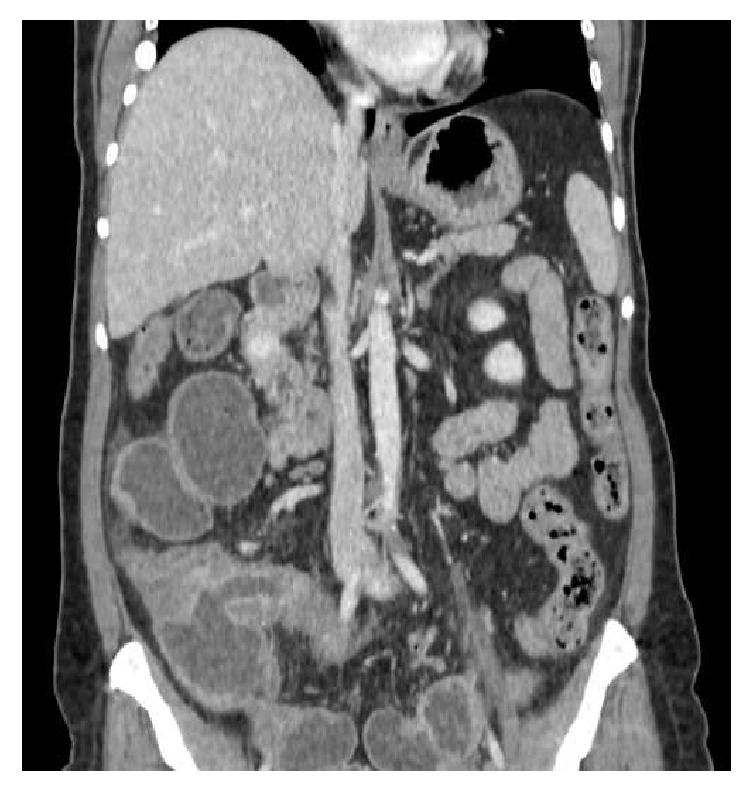


**Figure 2:**
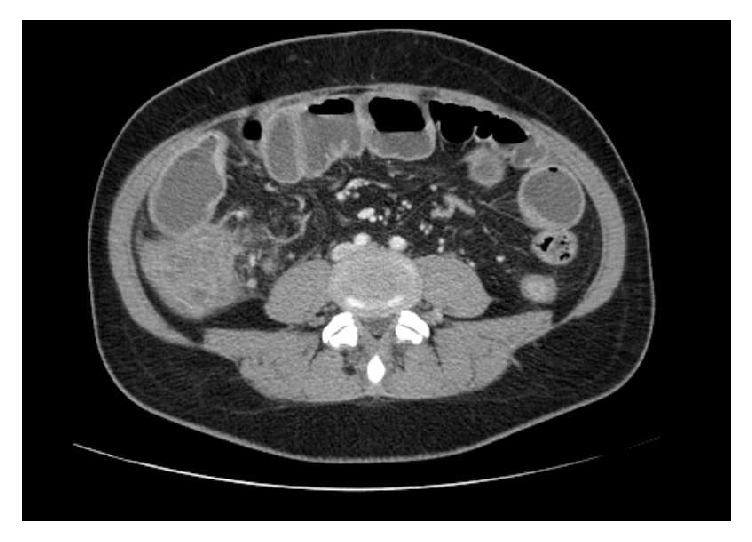


**Figure 3:**
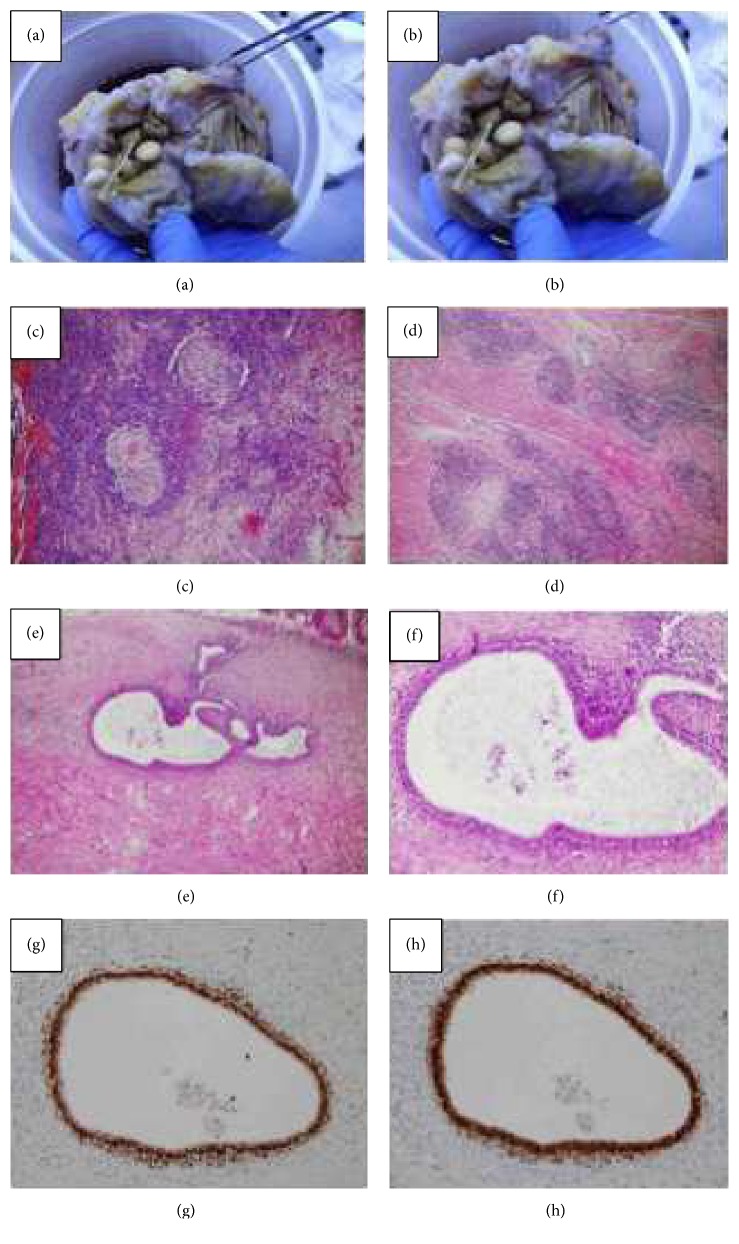
((a), (b)) Macroscopic features of the specimen with the stenotic area and mesalamine pills accumulation. ((c), (d)) CD with typical granuloma ((c) EE 4 HPF) and lymphoid aggregates ((d) EE 10 HPF). ((e)–(h)) Endometriosis focus ((e) EE 4 HPF; (f) EE 10 HPF) and immunohistochemical staining for estrogen ((g) 10 HPF) and progesterone ((h) 10 HPF).

A 45-year-old woman was admitted to our surgical department for the first time in November 2015, suffering from diffuse abdominal pain for over one month. She was also in amenorrhea without any other gynecologic symptoms. From 1988 she took the HAART (Highly Active Antiretroviral Therapy) for HIV infection with regular count of CD4 lymphocytes (last determination of the CD4 lymphocytes was 1005 (25.9%) and HIV viremia was not detectable). She had never been pregnant and had no abdominal surgery in her history. Before the recovery, the patient underwent an outpatient colonoscopy that reported a nontransitable severe stricture of the ascending colon that did not allow the execution of biopsies; the perilesion biopsies did not reveal any morphological specific alteration. During the recovery a CT with contrast medium was performed and a thickening of the wall of the terminal ileum/ascending colon compatible with inflammatory or lymphoproliferative disease was found (Figures 1 and 2). The treatment was only medical with fasting, infusions, antibiotics, and mesalamine (800 mg t.i.d.). One month later, after therapy with mesalamine, another colonoscopy was performed and confirmed the persistence of the stenosis. The patient still suffered severe abdominal pain and vomit. She was admitted to our department, an urgent laparotomy and a right hemicolectomy were performed. Intraoperative mass of the ascending colon occluded the intestinal transit without any adhesions with the other abdominal organs and no signs of endometriosis were found. The postoperative course was regular without complications. She was discharged on the eighth postoperative day. The histological examination on the specimen was “Crohn's Disease with outbreaks of endometriosis.” CD10, usually positive in endometriosis, was used for histological diagnosis. The macroscopical appearance of the surgical specimen revealed a stenosis of the terminal ileum at the level of ileocecal valve with accumulation of pills of mesalamine in the distal ileum (Figures 3(a) and 3(b)). The histological evaluation revealed a typical CD with sectorial erosions, ulcerations, and inflammation of the mucosa with multiple lymphoid aggregates in the submucosa, muscularis propria, and serosa together with foci of endometriosis (estrogen and progesteron positive immunostain) (Figures 3(c)–3(h)).

The patient is now incorporated into a multidisciplinary team including Gastroenterologists, Infectious Diseases Specialists, and Gynecologists.

## 3. Discussion

Our case report talks about two specific correlations, first between CD and HIV infection and second between CD and endometriosis.

Despite several decades of investigation, the etiology and specific pathogenetic mechanism responsible for CD remain poorly defined. T lymphocytes, in particular CD4+, seem to play a key role in regulating mucosal T-cell response to bacterial antigens, which may lead to intestinal inflammation; indeed, there are several reports of CD symptoms which spontaneously improved following the HIV infection and a subsequent decline in CD4+ count [10–13].

Viazis et al. [14] demonstrated that the maintenance of remission was statistically different in patients with IBD infected with HIV compared to a matched control group of IBD patients without HIV. The time to first relapse was longer for IBD-HIV patients, suggesting a protective role of the HIV infection. In particular this could be attributable to the lower CD4+ count, since nonlymphopenic patients without immunosuppression seemed to relapse more often than those with immunosuppression.

A review of 7 cases reports IBD active course or IBD relapse in HIV patients under immunosuppression; on the contrary, some patients who have reconstituted their immune system with HAART can have a silent IBD [15]. In our case, the patient had a high CD4+ count in HAART, like an immunocompetent one; this can justify the active IBD course. On the other hand, IBD and endometriosis are immune mediated chronic inflammatory disorders affecting young women [16]. Both IBD and endometriosis are often in differential diagnosis in women with abdominal pain and a preoperative definite diagnosis is challenging and sometimes impossible [17]. Guadagno et al. investigated the histological correlation between CD and endometriosis and concluded that in women with known endometriosis histological IBD-like lesions, such as crypt distortion and mucosal surface alteration, should be considered as an epiphenomenon of endometriosis, not as a true IBD [18]. A Danish study demonstrated an increased risk of developing IBD among a large cohort of 37661 women with endometriosis and the risk was higher in women who had endometriosis verified by surgery [19]. The authors speculated that this association may be explained by shared immunological features between endometriosis and IBD or it may reflect a consequence of endometriosis treatment (in particular the oral contraceptives).

Yantiss et al. [20] reported a large series of 44 surgical cases with intestinal endometriosis; 11 (25%) had luminal strictures secondary to fibrosis and smooth muscle hyperplasia. A total of 19 (42%) had mucosal changes similar to chronic colitis/enteritis as seen in IBD, including villous blunting, branched crypts or crypts distortion, deep lymphoid aggregates and fissures. This study demonstrated that intestinal endometriosis mimics gross and histologic finding of IBD.

Recently, an American study [21] investigated the association and clinical significance of concomitant endometriosis and IBD (51 cases of female IBD patients with endometriosis and 102 controls of female IBD patients without endometriosis). They compared the IBD phenotype and natural history, specifically the use of immunosuppressive and/or biologic therapy and the need for IBD-related surgery, among IBD patient with and without endometriosis. Among endometriosis-CD patients whose endometriosis was surgically verified, there was a higher rate of stricturing disease than CD controls, while there was no difference in natural history of IBD in terms of immunosuppressive therapy and IBD surgery.

In conclusion, the HIV infection seems to have a protective role in the behaviour of the IBD, in particular for the relapse. Endometriosis and IBD can occur simultaneously [22–24] maybe because of the potential mechanisms of disease pathogenesis like immune dysregulation or due to the medical endometriosis therapy (oral contraceptive). The differential diagnosis is difficult for the similar clinical presentation and is often only made after surgical resection of the diseased segment. It seems that CD patients with endometriosis have a high risk to develop structuring CD as in our case report.
